# Influences of Sensor Placement Site and Subject Posture on Measurement of Respiratory Frequency Using Triaxial Accelerometers

**DOI:** 10.3389/fphys.2020.00823

**Published:** 2020-07-09

**Authors:** Stephen Hughes, Haipeng Liu, Dingchang Zheng

**Affiliations:** ^1^Medical Devices Research Group, Anglia Ruskin University, Chelmsford, United Kingdom; ^2^Faculty Research Centre for Intelligent Healthcare, Coventry University, Coventry, United Kingdom

**Keywords:** accelerometer, respiration rate, respiration frequency (RF), chest wall, posture, sensor placement

## Abstract

**Introduction:**

Respiration frequency (RF) could be derived from the respiratory signals recorded by accelerometers which detect chest wall movements. The optimum direction of acceleration for accurate RF measurement is still uncertain. We aim to investigate the effect of measure site, posture, and direction of acceleration on the accuracy of accelerometer-based RF estimation.

**Methods:**

In supine and seated postures respectively, respiratory signals were measured from 34 healthy subjects in 70 s by triaxial accelerometers located at four sites on the body wall (over the clavicle, laterally on the chest wall, over the pectoral part of the anterior chest wall, on the abdomen in the midline at the umbilicus), with the reference respiratory signal simultaneously recorded by a strain gauge chest belt. RFs were extracted from the accelerometer and reference respiratory signals using wavelet transformation. To investigate the effect of measure site, posture, and direction of acceleration on the accuracy of accelerometer-based RF estimation, repeated measures multivariate analysis of variance, linear regression, Bland-Altman analysis, and Scheirer-Ray-Hare test were performed between reference and accelerometer-based RFs.

**Results:**

There was no significant difference in accelerometer-based RF estimation between seated and supine postures, among four accelerometer sites, or between seated or supine postures (*p* > 0.05 for all). The error of accelerometer-based RF estimation was less than 0.03 Hz (two breaths per minute) at any site or posture, but was significantly smaller in supine posture than in seated posture (*p* < 0.05), with narrower limits of agreement in Bland-Altman analysis and higher accuracy in linear regression (*R*^2^ > 0.61 vs. *R*^2^ < 0.51).

**Conclusion:**

Respiration frequency can be accurately measured from the acceleration of any direction using triaxial accelerometers placed at the clavicular, pectoral and lateral sites on the chest as well the mid abdominal site. More accurate RF estimation could be achieved in supine posture compared with seated posture.

## Introduction

Measurement of respiratory rate (RR) is defined as the cycles of breathing in 1 min, with breath per minute (bpm) the common unit. RR can be directly calculated from the respiratory frequency (RF) which is widely used in healthcare monitoring: RR (bpm) = RF (Hz)/60. RR is important to the delivery of safe medical care. A raised RR precedes clinical deterioration in many acute medical conditions (Hodgetts et al., 2002) and therefore its accurate measurement is of prime importance to patient safety.

Numerous studies have indicated that RR is not measured reliably in many cases (Granholm et al., 2016; Badawy et al., 2017). Currently, the commonest method of measuring RR in non-critical care settings is manual measurement in which a healthcare worker counts the number of breaths taken within a certain time period. However, the manual method has been proven liable to observer bias and errors, and is often conducted improperly or omitted (Cretikos et al., 2008). To yield patient safety benefits, there is an urgent clinical need for a device to achieve reliable RR (or RF) measurement.

Microelectromechanical systems (MEMS) accelerometer is a low-cost technology that could reliably record the chest wall movement during respiration, deriving the respiratory signals in three directions from which the RF can be derived (Bates et al., 2010). Currently, different algorithms have been developed to extract RF from accelerometer signals. However, the accelerometer signals derived in different body sites and postures are highly different in signal quality, waveform, and other properties (Vehkaoja et al., 2015). In many clinical settings such as primary care, outpatient clinic, and emergency department settings, vital sign measurements are undertaken as single intermittent measurements on patients at rest. Toward the reliable estimation of RF in different application scenarios such as driving, sleep, and daily activities (Liu et al., 2019), it is important to investigate the effects of different physiological factors (measurement site, posture, direction) on the RF estimation results. Therefore, it is worthwhile to explore the optimum settings for accelerometers to achieve high accuracy in a single RF measurement.

A clinically useful accelerometer-based respiratory device ought to use a minimum number of sensors in contact with the patient. From this arises the question as to be optimum site for deployment of the sensor. Pitts et al. (2013) found that respiratory waveforms obtained from sensors placed on the clavicle were more consistent and of higher amplitude than those obtained from sensors placed on the lower third of the sternum. They also found that a more reliable RR could be obtained from the clavicular site, particularly in the orthogonal *Z*-axis. In contrast, Siqueira et al. (2019) found that waveforms of greater amplitude and consistency were from sensors placed on the right side of the abdomen and the right costal margin in comparison with corresponding sites on the left side. However, they did not deploy sensors above nipple level. The clavicular site may seem attractive for development of a clinical device because the sensor may easily be placed under clothing and removed without too much intrusion. A comprehensive comparison of the effectiveness of accelerometer-based RR estimation between clavicular and other sites on the body wall was therefore conducted.

Motion artefact is a major factor that influences the quality of respiratory signals recorded by accelerometers and the accuracy of RF estimation. The intensity of motion artefact depends on the direction of acceleration, measurement site, and body posture. Posture influences chest wall motion and pulmonary function (Lee et al., 2010). Patients attending an emergency department tend to be either seated in an erect posture or recumbent in a supine posture. It is hypothesised that if posture significantly alters chest wall movement patterns then it will influence the optimum placement of the accelerometer sensor for RF measurement.

To achieve clinically reliable RF estimation by accelerometer at optimum site, the aim of this research was to compare the effectiveness of various accelerometer placement sites for RF measurement in resting adult subjects in postures that might be encountered in the triage room of an emergency department.

## Materials and Methods

### Subjects

Between the accelerometer and the strain gauge reference, a difference of 0.033 Hz in RF measurement (2 bpm in RR) is clinically significant. Accordingly, a sample size calculation was undertaken and a study group of no less than 31 participants was found to be necessary to achieve 90% power. Ethical approval for the study was obtained from the Faculty Research Ethics Panel of the Faculty of Medical Science at Anglia Ruskin University, Chelmsford, United Kingdom.

We recruited 34 subjects (18 male, 16 female). Each was screened for good health by means of a questionnaire and exclusion criteria including age >60 years, cardiac or respiratory disease, current medication use, and pregnancy. Racial demographic data was not collected however, our subject group included participants from a range of ethnic groups including multiple people of Caucasian, South Asian, West African and Chinese origin. All participants were able to complete the measurement runs with no adverse effect reported. Their demographic characteristics and mean resting RRs are set out in Table 1. The RR values covered the range of normal adult RR (12–20 bpm).

**TABLE 1:** Demographic characteristics of participants.

	**Mean**	**Min**	**Max**	***SD***
Age (years)	26	18	54	8.5
Height (cm)	173.8	155	193	10.9
Weight (kg)	72.6	48	97	19.9
RR Seated (bpm)*	13.40	5.86	24.61	4.19
RR Supine (bpm)*	12.52	4.69	24.61	4.55

*There is no significant difference between RR Seated and RR Supine (p > 0.05).

### Data Collection

**FIGURE 1:**
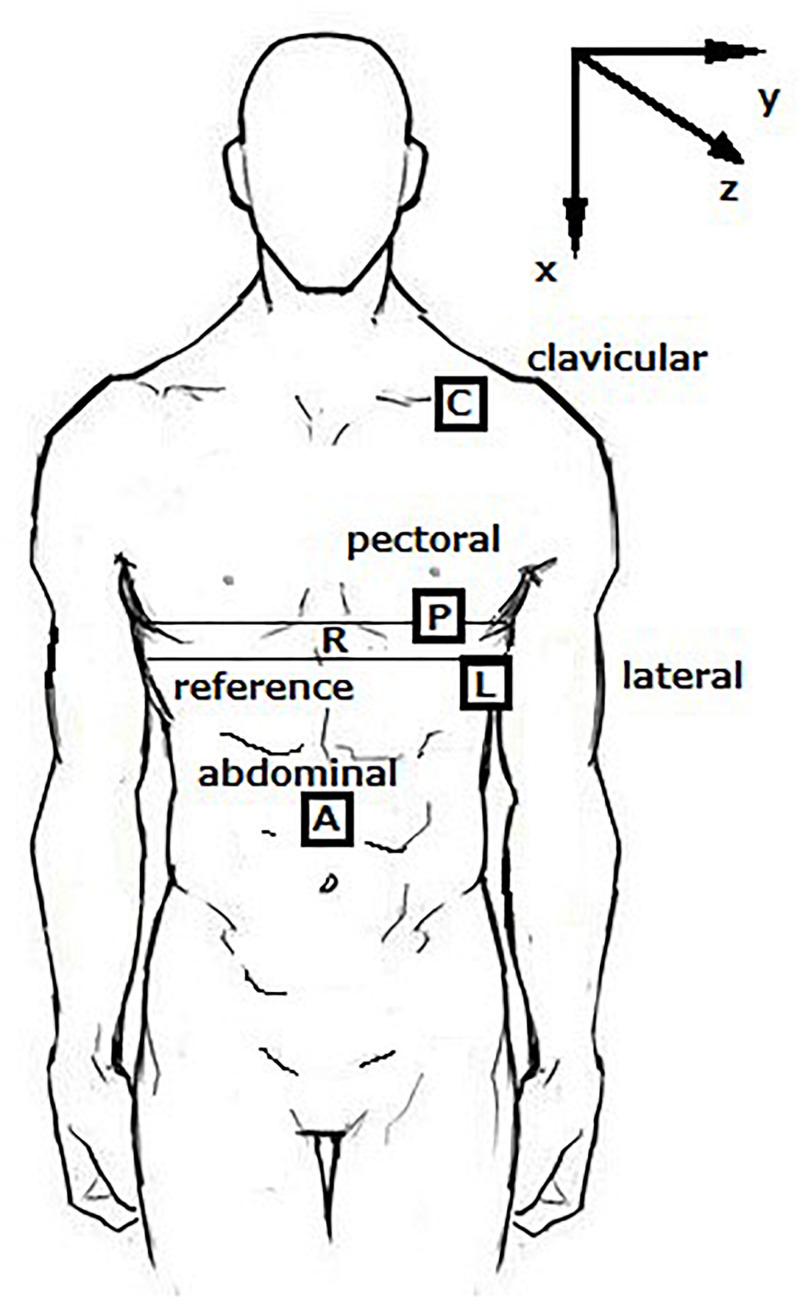
Sensor Placement Sites on the body and Orthogonal Axes.

Analogue MEMS 5G accelerometers [TSD109C2 (±5G), Biopac Systems, Goleta, CA, United States] were placed on four sites of the body, as shown in Figure 1. The bandwidth of the accelerometer is 500 Hz. The BIOPAC accelerometer has been fully validated and used as the reference sensor in the development of medical devices (Costa and Fazendeiro, 2014; Aguilera-Castells et al., 2020; Jacunski and Rafferty, 2020). The dimensions are 16 mm long, 17 mm wide, and 8 mm high, which are appropriate for the simultaneous measurement in different body sites. Three sensors were placed on the left side of the chest and one was placed on the abdomen. These sites were:

(i) Clavicular: over the lateral third of the clavicle.
(ii) Pectoral: the pectoral region of the chest at level of the fourth interspace in the midclavicular line.
(iii) Lateral: at the same level as the pectoral accelerometer but in the mid-axillary line.
(iv) Abdominal: at the level of the umbilicus.

Each device was mounted on the body with fabric straps and secured on to the straps with Velcro. Each device had been calibrated immediately prior to commencement of the study in accordance with the manufacturers’ instructions. The sampling frequency was 500 Hz.

The reference device for RF measurement was a Biopac SS5LB strain gauge device circumferentially fitted on a fabric belt and mounted on the chest during measurement (the “R” belt in Figure 1). Outputs from strain gauge and the accelerometers were collected simultaneously using Biopac MP160 data acquisition system and saved by the Acqknowledge^®^ software. The analogue signals from the reference device and the accelerometers were DC filtered in hardware by the MP160 data acquisition device. The RF extracted from the stain gauge signal was used as the reference value to evaluate the accuracy of RF values derived by accelerometers.

Each participant breathed normally during the measurement. Experimental runs were performed in supine and seated postures in randomised order. There were three repeated runs in each posture. There was a gap of 2 min between measurement runs when the posture, attachment of all devices, and the reliability of collected data were checked.

### Signal Processing

**FIGURE 2:**
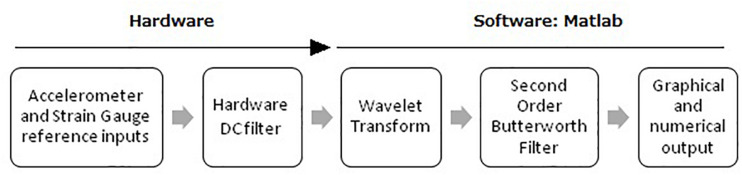
Signal Processing Algorithm. DC, direct current.

Further signal processing was implemented in Matlab (Mathworks Inc. Natick, MA, United States). Figure 2 describes the signal processing algorithm. The DC-filtered signals were firstly processed using a wavelet algorithm. For frequency domain analysis (e.g., PSD), the variation of RF during the measurement can cause estimation error (Hartmann et al., 2019). To improve the reliability of RF estimation we used wavelet analysis which discloses the properties of the signals in time-frequency domain. Daubechies wavelets have moderately smooth shape and the oscillating pattern is similar to the shape of heartbeat and respiration pattern buried in accelerometer signals under different scales, which makes it suitable for RF extraction (Lin and Jhou, 2020). Therefore, the accelerometer signals were decomposed using Daubechies wavelet with 6 vanishing moments (db6) at the level 9. The results were transformed to frequency domain and further processed by a second order low-pass Butterworth Filter with cutoff frequency at 0.9 Hz.

The 0.9 Hz low pass cutoff was determined retrospectively after all data had been collected and was found to be the optimum cutoff across a RF range of 0.1–0.5 Hz (6–30 bpm in RR) which covers the RRs that might be encountered clinically. The RF was determined as the frequency of peak intensity in frequency domain.

### Statistical Analysis

Data were analysed in SPSS (Version 24.0, IBM Corp). To investigate the repeatability of three measurements, as well as the effects of sensor placement site, direction of acceleration, and posture on the RF measurement results, repeated measures multivariate analysis of variance (MANOVA) was firstly performed. Mauchly’s sphericity test and Levene’s test were performed to investigate if sphericity (defined as *p* more than 0.05) and homoscedasticity (or homogeneity of variance, defined as p more than 0.05) were satisfied. If sphericity and homoscedasticity were not satisfied, the non-parametric Scheirer-Ray-Hare test was performed on two factors each time with R language (R Core Team, 2018). In MANOVA and Scheirer-Ray-Hare test, the least significant difference (LSD) *post hoc* multiple comparisons and Dunn’s Kruskal-Wallis multiple comparisons were, respectively, performed for *post hoc* analysis. Significant difference was defined as *p* < 0.05.

**FIGURE 3:**
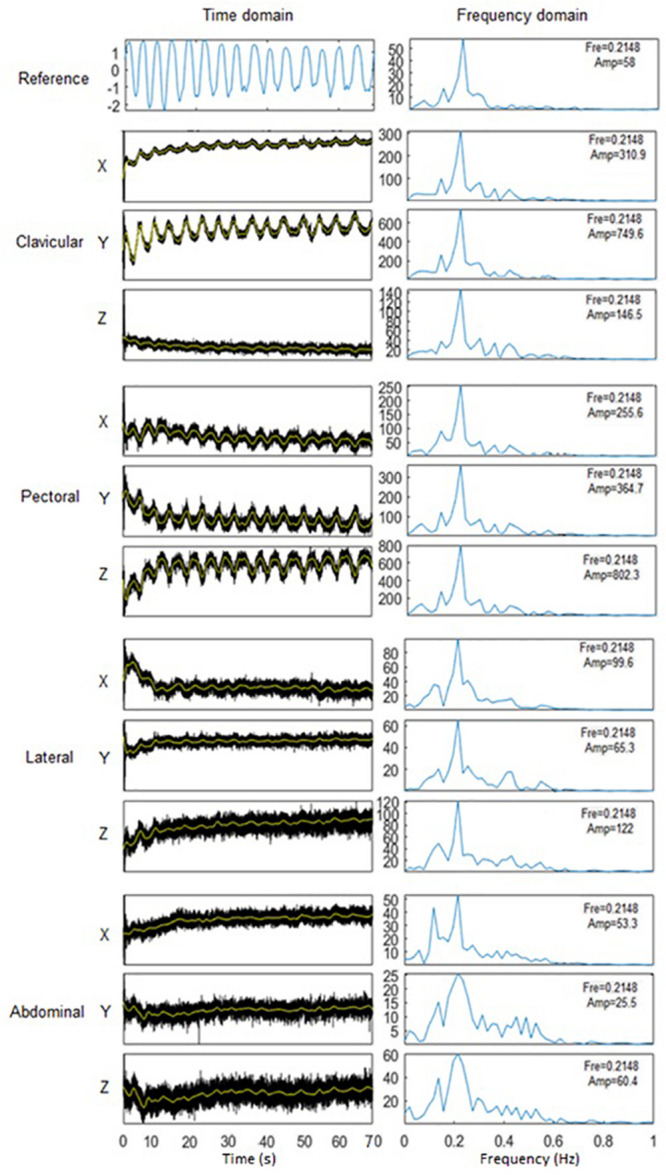
Extraction of respiratory frequency (RF) in Matlab. Left: waveform of processed respiratory signals in time domain. Signals in 3 directions in 4 sites were juxtaposed to give an indication of signal quality. Right: signal intensity spectra in frequency domain. RF was estimated by as the peak frequency (Fre), with the amplitude (Amp) of signal intensity marked.

**FIGURE 4:**
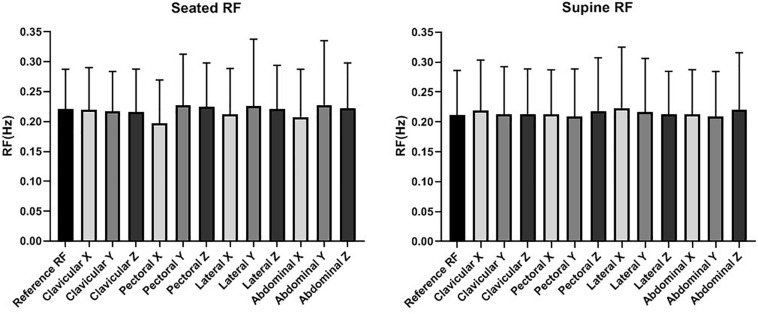
Respiration frequency (RF) measurement in seated and supine postures at different sites.

The error of accelerometer-based RF estimation was calculated as shown in Figures 3, 4, respectively, and the difference between RF values measured by accelerometer and reference strain gauge. For each measurement, the difference value was normalised by the corresponding reference RF value. Based on the results that there was no significant difference in RF estimation among three repeated measurements (see section Results: RF Estimation), for the MANOVA or Scheirer-Ray-Hare test, RF estimation error was calculated as the averaged value of the three normalised differences. Considering the existing of zero values, besides Levene’s test, the Shapiro-Wilk test was performed to investigate if the data followed normal distribution.

Bland-Altman analysis was performed to illustrate the distribution of RR estimation errors. Finally, linear regression analysis was performed to investigate the consistency in RR estimation between the accelerometer and stain gauge.

## Results

### RF Estimation

The results in Mauchly’s sphericity test (*p* > 0.05) and Levene’s test (*p* > 0.05) showed that repeated measures MANOVA was reasonable. The results indicated no significant difference between the three measurements, four sites (clavicular, pectoral, lateral, abdominal), three directions (x, y, z), and two postures (seated, supine) (*p* > 0.05 for all).

### Estimation Error of RF by Accelerometer

The RF estimation error did not follow normal distribution (*p* < 0.05 in Shapiro-Wilk test for all) or homoscedasticity (*p* < 0.001 in Levene’s test). Therefore, three Scheirer-Ray-Hare tests were performed based on the posture-direction, direction-site, and site-posture pairs, respectively. The results showed that the RF estimation error was only significantly influenced by posture and its interaction with direction (both *p* < 0.05). The results of Dunn’s Kruskal-Wallis multiple comparisons showed that the errors derived in supine posture are significantly smaller than those derived in seated posture (*p* < 0.05).

### Bland-Altman Analysis

**FIGURE 5:**
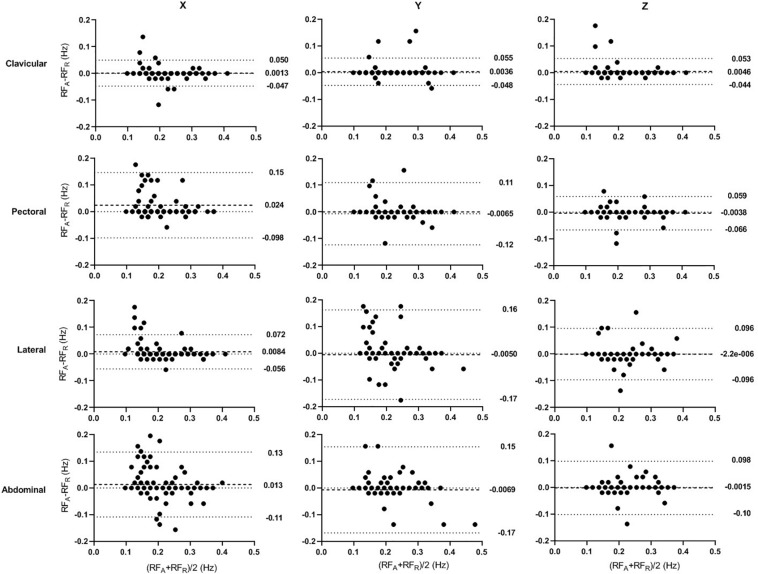
Bland–Altman Plots of the accelerometer-derived and reference RFs in seated posture. The *x*- and *y*-axes indicate the mean and difference of the accelerometer-derived and the corresponding reference RFs, respectively. The dashed lines show the mean bias. The limits of agreement (LoA) mean ± 1.96 *SD* interval of difference are given with the dotted lines.

**FIGURE 6:**
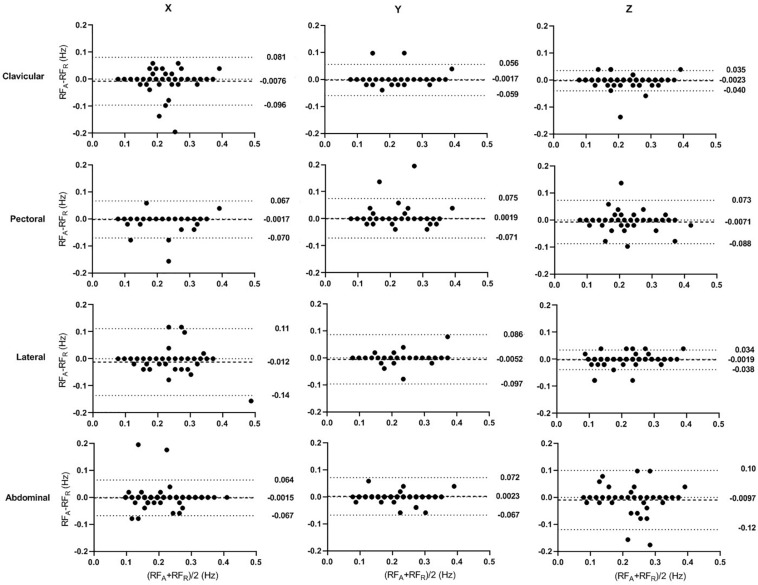
Bland–Altman Plots of the accelerometer-derived and reference RFs in supine posture. The *x* and *y*-axes indicate the mean and difference of the accelerometer-derived and the corresponding reference RFs, respectively. The dashed lines show the mean bias. The limits of agreement (LoA) mean ± 1.96 *SD* interval of difference are given with the dotted lines.

The Bland-Altman analysis of accelerometer-derived and reference RFs in seated and supine postures are shown in Figures 5, 6, respectively. Compared with results of seated posture, the supine posture showed lower bias and narrower limits of agreement (LoA). In the seated posture, when compared with other sites, the clavicular site has the narrowest LoA in all three directions. Whereas, in the supine posture, different sites are comparable in bias and LoA. The best results are from the lateral site in z direction (bias: -0.0019 Hz, LoA:-0.038 to 0.034 Hz), followed closely by the result of clavicular size in z direction (bias: -0.0023 Hz, LoA: -0.040 to 0.035 Hz). The results are in accordance with aforementioned results that posture has an effect in significantly on the error of accelerometer-based RF estimation.

### Linear Regression Analysis

**FIGURE 7:**
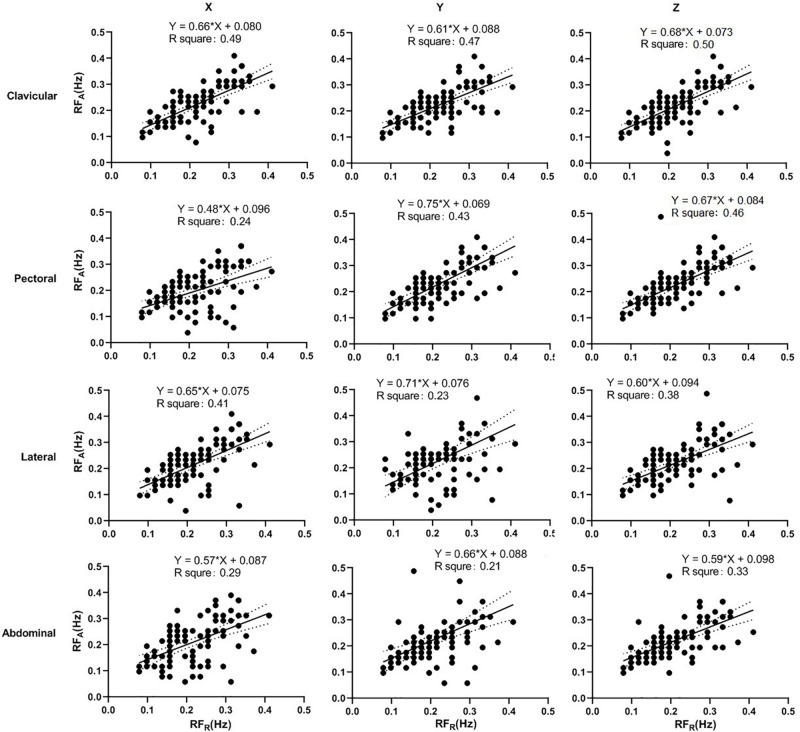
Linear regression of the accelerometer-derived and reference RFs in seated posture. The *x*- and *y*-axes denote the reference and accelerometer-derived RFs, respectively. The points mark the measured data. The solid and dotted lines indicate the best-fit line and its 95% confidence interval, respectively.

**FIGURE 8:**
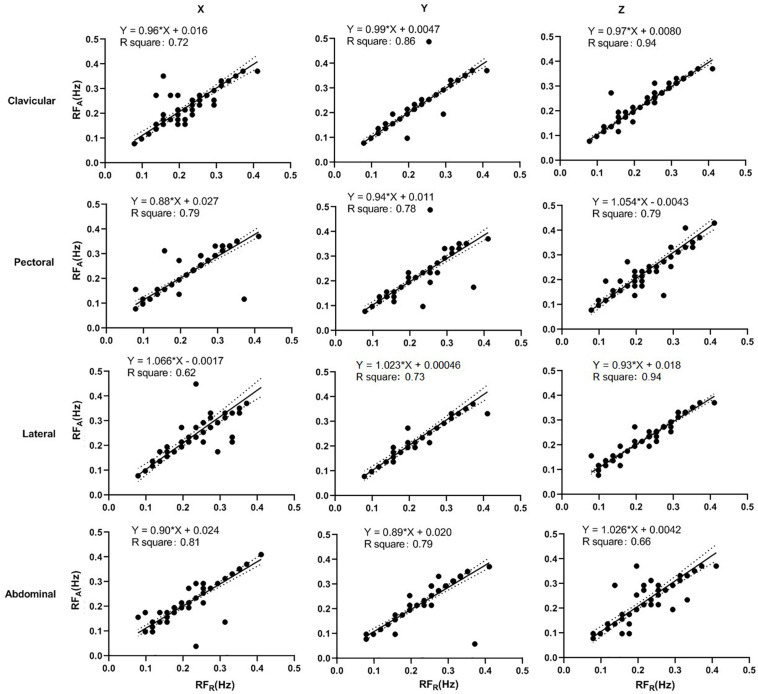
Linear regression of the accelerometer-derived and reference RFs in supine posture. The *x*- and *y*-axes denote the reference and accelerometer-derived RFs, respectively. The points mark the measured data. The solid and dotted lines indicate the best-fit line and its 95% confidence interval, respectively.

The linear-regression analysis of accelerometer-derived and reference RFs in seated and supine postures are shown in Figures 7, 8, respectively. The distribution of data points are obviously closer to the regression line in the supine posture. In seated posture, the results derived in different sites and directions are comparable. In the supine posture, The result from clavicular site in z direction derived the highest *R*^2^ in both seated (0.50) and supine (0.94) postures, indicating a high linearity of data distribution.

## Discussion

### Effect of Measurement Site and Acceleration Direction on the Accuracy of RF Estimation

Recently, various types of sensors such as piezoelectric sensor, microwave radar, and thermal imaging have been proposed for non-contact respiratory monitoring (Liu et al., 2019). In comparison, the accelerometer is a low-cost, reliable, and convenient sensor that is promising for the wearable respiratory monitoring. Our results suggest that triaxial accelerometers can be used to measure respiration rate in both the seated and supine postures. Recently, Lin and Jhou used wavelet analysis to extract RF from the seismocardiogram signal recorded by the MEMS triaxial accelerometers in supine posture, with the estimation error of 0.0035 ± 0.0628 Hz (0.21 ± 3.77 bpm in RR) (Lin and Jhou, 2020). The authors concluded that the accelerometer could be a potential tool for RR monitoring. In our results, there was no significant difference in the RF measured at the sites that we employed or between the orthogonal planes (directions of accelerations). In all the measurement sites and orthogonal planes, the error in RF estimation was within the range of ± 0.033 Hz (±2 bpm in RR) that was thought to be clinically significant. Similarly, Drummond et al. applied the triaxial accelerometers in the RR monitoring for postoperative subjects, and found that the instantaneous RR matched within 2 bpm on 86% of occasions (Drummond et al., 2011). Our results suggest that RF can be reliably measured from any of the sites on the body wall that we employed in this study.

It has been proven that with a gap longer than 10 min, there was significant difference (>30% of measured value) between repeated measurements of RF (Edmonds et al., 2002). In our parallel study, with the gap of 1 min, there was no significant difference between repeated measurements of RF (Al-Halhouli et al., 2020). Therefore, we used the gap of 2 min to ensure the repeatability of the RF measurements while the subjects could have a good rest.

Despite the insignificance in statistical analysis, there are some observable differences between sites and directions. In Bland-Altman analysis, the clavicular site has the least bias and narrowest LoA compared with other sites, except in x direction of supine posture. In linear regression analysis, the clavicular site either has the slope most approximate to 1 and highest *R*^2^, or has the values comparable with the best site. The RF estimation from z direction has the least bias and narrowest LoA, with the highest *R*^2^ in linear regression analysis, at all the sites except lateral in seated posture, and at clavicular and lateral sites in supine posture. It has been suggested that the acceleration in z direction is the most sensitive to the thoracic movement (Kwon et al., 2011). Therefore, the best results seem in general, to arise from the clavicular site in z-axis in both the seated and supine postures.

Our finding that the clavicular site gives acceptable correlation with reference is consistent with that of Pitts et al. (2013) and we confirm this using a larger and more statistically significant sample size. Additionally, we demonstrate that the clavicular site performs well in both seated and supine postures, whereas Pitts’ subjects were all seated.

The results of Siqueira et al. (2019) demonstrate as we do that different sites on the body wall give viable respiratory waveforms that differ in amplitude. Their group combined accelerometer outputs using independent component analysis and demonstrated that the use of more accelerometer sites in certain combinations decreases the mean squared error between device and reference outputs and therefore increases the reliability of RF measurements. Siquiera et al. chose sites around the thoracic and abdominal areas including the lateral abdominal site while we included the clavicular site. Furthermore, our participants included a high proportion of female subjects (47.1%) whereas those of Siquiera’s group had none.

### Effect of Posture on the Accurate of RF Estimation

The results of statistical analysis, Bland-Altman analysis, and linear regression analysis commonly indicate that supine posture is significantly more accurate than seated posture in RF estimation.

The difference between postures may be accounted for by increased body movement when seated, the supine posture being more relaxed and stable and not requiring corrective postural movements to stay erect. It has been proven that higher accuracy in the measurement of both heart rate and respiratory rate could be achieved in supine posture compared with in sitting posture, using either accelerometer or gyroscope (Hernandez et al., 2014). As aforementioned, the accelerometer measurement is highly sensitive to motion artefact.

A parallel study on the extraction of heart rate by accelerometer showed supine posture could derive more accurate detection of peak-to-peak intervals compared with sitting posture (Kwon et al., 2011). A recent study showed that the error in RF estimation from the in-ear accelerometer signals is much higher in standing and sitting postures compared with supine posture (Röddiger et al., 2019). Therefore the measurement of RF in supine posture could achieve more reliable and robust RF estimation results.

### Clinical Application

We have demonstrated that a single sensor may be placed almost anywhere on the chest wall to obtain a respiratory signal. The results provide an important reference for the related study in clinical and daily RF monitoring. A clinically applicable system should ideally use the minimum number of sensors in contact with a patient, for reasons of patient acceptability, ease of use and manufacturing cost. To be applied in wearable sensors, low consumption of computing resources is an important consideration. Our signal algorithm is basic and appears to cater well for measurement of RR by a single sensor because the outputs of multiple sensors are not combined. This may find application in the development of respiratory rate measurement devices for clinical use. Some advanced algorithms, such as those based on the autocorrelation model, could improve the accuracy of short-time RF estimation using accelerometers in clinical applications, which deserves further investigation (Sun and Matsui, 2015).

The clavicular site is readily and conveniently accessible on patients. Our results disclosed that clavicular is suitable for reliable RF measurement, which may find application in the development of a clinically deployable RR measurement system.

Considering the degrading of accelerometer signal caused by motion artifact (Preejith et al., 2017), it is likely that accelerometers are less suitable for long-term RR monitoring in postures other than supine. However, accelerometers may be suitable for single measurements, such as those commonly taken in primary care and the triage areas of hospital emergency departments.

### Limitations and Future Direction

A number of outliers were identified in our data with large data biases. Their occurrence appeared sporadic and unrelated to individual subjects. One way in which these may be explained by a failure of securing of the accelerometers to the body wall. If that is the case then our results might be improved by fixing the accelerometers directly to the skin with tape. Our system used fabric straps and Velcro. This is less mechanically sound than direct fixation to the skin, however, it may provide a better simulation of the clinical situation where not all staff and patients in an ambulatory care or emergency department triage situation may find it acceptable to affix the sensors with skin tape (Schneller et al., 2017). Further work into the mechanical coupling of the sensors is necessary.

Another source of inaccuracy may arise from subjects not breathing regularly or naturally. One subject whose results were outliers had a low resting RR and was noted to have a significant interval between inhalation and exhalation in effect, a breath holding. This may allow noise from extraneous movement to contaminate the accelerometer signal.

We found no effect of gender on the results. This suggests that a device employing accelerometers to measure respiration rate may be equally used on males and females, however, more participants of each gender are required to confirm this.

## Conclusion

Reliable measurement of RF can be obtained from a single MEMS triaxial accelerometer sensor at a number of body wall sites on the chest and the mid-abdomen, in all the three direction, and in both supine and sitting postures. Compared with seating posture, supine posture has higher accuracy.

## Data Availability Statement

The datasets generated for this study are available on request to the corresponding author.

## Ethics Statement

The studies involving human participants were reviewed and approved by the Faculty Research Ethics Panel, Faculty of Medical Science, Anglia Ruskin University. The patients/participants provided their written informed consent to participate in this study.

## Author Contributions

SH and DZ conceived the study and collected the data. SH and HL analyzed the data and drafted the manuscript. All authors were involved in the discussion of the results and revision of the manuscript.

## Conflict of Interest

The authors declare that the research was conducted in the absence of any commercial or financial relationships that could be construed as a potential conflict of interest.
